# Subcutaneous adipose tissue measured by computed tomography could be an independent predictor for early outcomes of patients with severe COVID-19

**DOI:** 10.3389/fnut.2024.1432251

**Published:** 2024-10-14

**Authors:** Weijian Zhou, Wenqi Shen, Jiajing Ni, Kaiwei Xu, Liu Xu, Chunqu Chen, Ruoyu Wu, Guotian Hu, Jianhua Wang

**Affiliations:** ^1^Department of Radiology, The First Affiliated Hospital of Ningbo University, Ningbo, Zhejiang, China; ^2^Health Science Center, Ningbo University, Ningbo, Zhejiang, China; ^3^Department of Radiology, The First Affiliated Hospital of Xiamen University, Xiamen, Fujian, China

**Keywords:** computed tomography, subcutaneous adipose tissue, prognostic nutrition index, COVID-19, prediction

## Abstract

**Background:**

Patients with severe Coronavirus Disease 2019 (COVID-19) can experience protein loss due to the inflammatory response and energy consumption, impairing immune function. The presence of excessive visceral and heart fat leads to chronic long-term inflammation that can adversely affect immune function and, thus, outcomes for these patients. We aimed to explore the roles of prognostic nutrition index (PNI) and quantitative fat assessment based on computed tomography (CT) scans in predicting the outcomes of patients with severe COVID-19.

**Methods:**

A total of 130 patients with severe COVID-19 who were treated between December 1, 2022, and February 28, 2023, were retrospectively enrolled. The patients were divided into survival and death groups. Data on chest CT examinations following admission were collected to measure cardiac adipose tissue (CAT), visceral adipose tissue (VAT), and subcutaneous adipose tissue (SAT) and to analyze the CT score of pulmonary lesions. Clinical information and laboratory examination data were collected. Univariate and multivariate logistic regression analyses were used to explore the risk factors associated with death, and several multivariate logistic regression models were established.

**Results:**

Of the 130 patients included in the study (median age, 80.5 years; males, 32%), 68 patients died and 62 patients survived. PNI showed a strong association with the outcome of severe COVID-19 (*p* < 0.001). Among each part of the fat volume obtained based on a CT scan, SAT showed a significant association with the mortality of severe COVID-19 patients (*p* = 0.007). However, VAT and CAT were not significantly correlated with the death of patients. In the multivariate models, SAT had a higher predictive value than PNI; the area under the curve (AUC) of SAT was 0.844, which was higher than that of PNI (AUC = 0.833), but in the model of the combination of the two indexes, the prediction did not improve (AUC = 0.830), and SAT lost its significance (*p* = 0.069).

**Conclusion:**

Subcutaneous adipose tissue measured by computed tomography and PNI were found to be independent predictors of death in patients with severe COVID-19.

## 1 Introduction

The severe acute respiratory syndrome coronavirus 2 (SARS-CoV-2) is the pathogen responsible for COVID-19 (1, 2), which has spread widely throughout the world due to its highly contagious nature (3). The global outbreak of COVID-19 has posed a major threat to the health of humankind (4). Until 11th August 2024, there were 775,917,102 cases confirmed by COVID-19 and 3,742 death cases. During the 28-day period from 24 June to 21 July 2024, over 186,000 new cases and more than 2,800 new deaths, an increase of 30% and 26%, respectively, compared to the previous 28 days (27 May to 23 June 2024), and this data does not reflect a real number of new cases and new deaths, cause many countries have already stopped to report it (5).

As the illustration of Diagnosis and treatment protocol for COVID-19 patients (Tentative 10th Version) in China, severe COVID-19 refers to adults meeting any one of the following: a. shortness of breath, RR ≥ 30 times/min; b. in the resting state, the pulse oxygen saturation (SpO_2_) is ≤93% while breathing ambient air; c. arterial partial pressure of oxygen (PaO_2_)/the fraction of inspired oxygen (FiO_2_) ≤300 mmHg (6). Patients with comorbidities, such as diabetes, cardiovascular disease, and chronic respiratory diseases, were most likely to suffer severe illness and death (7–12). A common feature of patients with multiple comorbidities is poor nutrition, which may further perpetuate or aggravate the adverse consequences of various chronic diseases and exacerbate the unstable effects of diseases associated with acute inter-morbidity, such as COVID-19 diseases (13). For severe COVID-19 patients, prognostication is very important to the survival of patients because it can deeply impact the monitoring and interventional plan. Fried et al. (14) found that patients with severe COVID-19 could be discharged from the emergency room through closer monitoring methods, specific benchmarks for escalating care, strict inclusion criteria, and coordination of expectations with the patient.

The Prognostic Nutrition Index (PNI) is an indicator for the assessment of perioperative nutrition using the preoperative serum albumin level and total lymphocyte count (15). Previous studies have expressed concern about the loss of protein experienced by patients with severe pneumonia, resulting in damage to the immune defensive system (16). In patients with COVID-19, many factors, including an increased metabolic rate resulting from inflammation, increased energy expenditure due to mechanical ventilation, insufficient caloric intake, or muscle atrophy due to physical immobility (17, 18), can lead to deterioration of the nutritional status, especially in severe COVID-19 patients. A retrospective study performed showed that a lower PNI is related to a worse prognosis in severe COVID-19 patients (19). Al-Shami et al. found that PNI was significantly lower in ICU patients as compared to non-ICU patients (37.26 vs.41.29, *p* < 0.001) (20). Another study in Vietnam found that patients with malnutrition assessed by PNI have a greater proportion of severe and critical COVID-19 (21). Therefore, the ability of PNI to predict the outcome of patients with severe COVID is worth investigating.

Obesity was considered to be one of the risk factors for severe COVID-19 (22). Charpentier et al. suggested that cardiac adipose tissue (CAT) volume assessed on CT is an independent factor in predicting the outcome of COVID-19 patients with type 2 diabetes mellitus (23). A two-sample Mendelian randomization (MR) study found a causal association between higher visceral adipose tissue (VAT) and susceptibility, hospitalization, and severity of COVID-19 (24). Petrilli et al. recent meta-analysis indicated a link between visceral fat and increased COVID-19 severity, whereas subcutaneous fat (SAT) did not show the same association (25). Individuals with high levels of visceral fat may experience abnormal secretion of adipokines and cytokines like tumour necrosis factor-*α* and interferons, leading to chronic low-level inflammation. This inflammation can impair the immune response (26) and affect lung parenchyma and bronchi (27). Visceral fat contains an abundance of SARS-CoV-2 receptors and can thus become a “repository” of the virus, adversely affecting patient outcomes.

In addition, patients with severe COVID-19 can experience significant protein loss due to both inflammation and energy consumption, adversely affecting the patient’s immune function. At the same time, the presence of excess visceral and heart fat can lead to a long-term, low-grade inflammatory state, thus damaging the immune system and potentially inducing a cytokine storm following SARS-CoV-2 infection. Thus, we hypothesized that the assessment of the patient’s nutritional status, together with the quantitative measurement of the body fat content, can predict the outcomes of patients with severe COVID-19 and provide a reference for further clinical decision-making, improving patient outcomes and prognosis.

## 2 Materials and methods

### 2.1 Study population

This study has retrospectively included the patients admitted for COVID-19 (positive polymerase chain reaction (PCR) for SARS-CoV-2) between December 1, 2022, and February 28, 2023, in a large tertiary care academic central. The inclusion criteria in this study were: (1) a positive PCR test for SARS-CoV-2; (2) the presence of lung inflammation observed on chest CT examination; (3) clinical manifestations meeting the diagnostic criteria of severe COVID-19 according to the Diagnosis and Treatment Protocol for COVID-19 (Trial version 10) in China; (4) age ≥ 18 years. The exclusion criteria included: (1) poor quality chest CT imaging results (such as interference caused by motion and metal artifacts); (2) insufficient scan range of chest CT. The patients were divided into two groups according to outcome, namely, death and survivor.

### 2.2 Data collection

The blood analysis at admission includes white blood cell (WBC), neutrophil, lymphocyte and platelet counts, C-reactive protein (CRP), total bilirubin, D-dimer, creatinine, albumin, globulin, blood urea nitrogen (BUN), glomerular filtration rate (GFR), lactate dehydrogenase (LDH), glutamic pyruvic transaminase (GPT or ALT), glutamic oxaloacetic transaminase (GOT or AST), Creatine Kinase-MB (CK-MB) and interleukin 6 (IL-6). PNI has been widely applied in hospitals to reflect patients’ immunological and nutritional status (28). This tool is calculated by the following equation: PNI = [10 × serum albumin (g/dL)] + [0.005 × total lymphocyte count (mm^3^)](29). The WBC neutrophil, lymphocyte, C-reactive protein (CRP), and IL-6 are associated with the severity of inflammation; total bilirubin, LDH, AST, ALT, and CK-MB can reflect the function of the liver; BUN, GFR, and creatinine reflect the renal function; albumin, globulin, and PNI present the nutritional status.

A multidetector chest CT was performed upon admission to evaluate the severity of the COVID-19 pneumonia. This study would use CT imaging to measure the volume of cardiac adipose tissue (CAT), visceral adipose tissue (VAT), and subcutaneous adipose tissue (SAT).

All demographic, clinical, and laboratory data were collected from the digital records after hospital admission and during the clinical course. A specialized ICU physician collected and recorded these data, and two professional radiologists performed adipose tissue segmentation and volumetry on chest CT images of the patient. The outcome endpoint was a composite measure that included death or survival at discharge. All the data will be recorded using Microsoft Excel software.

### 2.3 CT acquisition protocol

For all the patients with COVID-19, a chest CT scan would be performed within 24 h of admission with one of three scanners (128-slice Precision, CAMPO IMAGING, 256-slice Brilliance, Philips Healthcare, 16-slice Apsaras, KANGDA). The patient was in the supine position during inspiration. The scanning parameters were a tube voltage of 120 kV, an automatic tube current modulation or tube current of 50 mA, a collimation width of 0.625 mm, an acquisition slice thickness of 5.0 mm, and an interval of 5.0 mm. Acquisitions were performed with or without the injection of contrast media.

### 2.4 Evaluation of the lung severity score from CT imaging

Currently, the chest CT scan is considered the most effective way to detect and assess the severity of abnormality in the lung (30). The severity of COVID-19 is strongly associated with the prognosis of patients (31), and Emanuel et al. also proposed the use of the severity score to assess the severity of CT findings in the chest (32). In this study, a semi-quantitative scoring system based on CT imaging was used to assess the severity of abnormality in the lung (33, 34). This was based on the extent of the lesions in each lung lobe (range, 0–4), the highest CT values of the lesion (range, 0–5), the presence or absence of pleural effusion (range, 0–3), and the presence or absence of mediastinal lymphadenopathy (range, 0–3). The total score was the sum of each lobar severity score, the highest CT value score of lung lesion, pleural effusion score, and mediastinal lymphadenopathy score, which was shown in Table 1.

**Table 1:** Details of the scoring system for the severity of pulmonary involvement from CT scores.

Score	Definition
The extension of lesions in each lung lobe	
0	None of lobe
1	1–25% of lobe
2	25–50% of lobe
3	50–75% of lobe
4	75–100% of lobe
The highest score of five lobes is 20	
The highest CT value of lung lesions	
1	≤−600HU
2	>−600HU and ≤ −400HU
3	>−400HU and ≤ −200HU
4	>−200HU and ≤ 0HU
5	>0HU
Pleural effusion	
3	Yes
0	No
Mediastinal lymphadenopathy	
3	Yes
0	No

All CT images were reviewed in random order by six radiologists who were blinded to the baseline and clinical information of the participants. The six radiologists independently assessed the CT features using axial images. Images were interpreted at a window of 1,000 to 2,000 Hounsfield Units (HU) and a level of −700 to −500 HU, respectively, to assess the lung parenchyma.

### 2.5 Assessment of CT fat deposition

**Figure 1:**
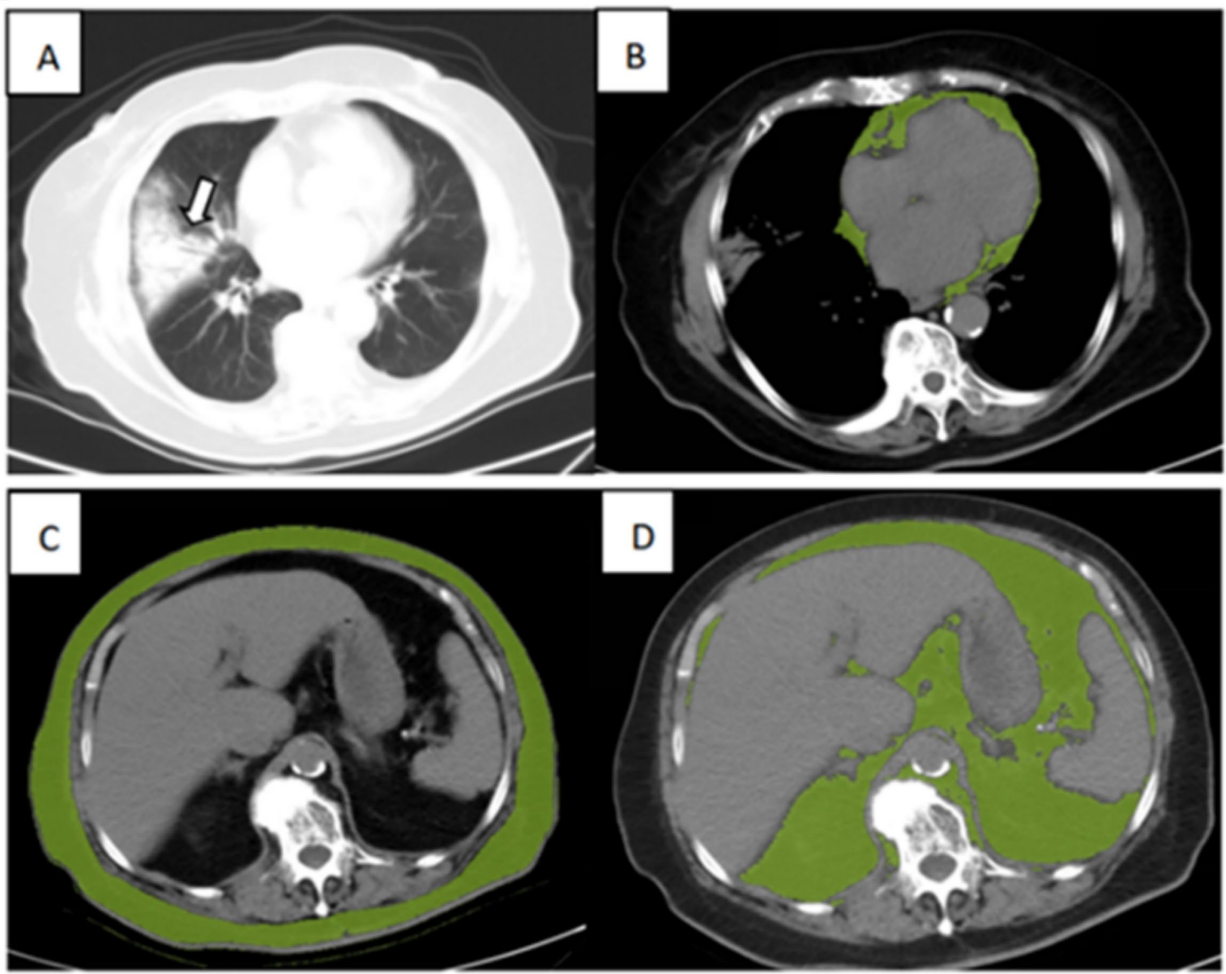
Illustration of adipose tissue volume assessed by the semi-automated segmentation method. **(A)** Chest CT for lung severity, **(B)** Cardiac adipose tissue (CAT), **(C)** subcutaneous adipose tissue (SAT), and **(D)** visceral adipose tissue (VAT) of an 89-year-old surviving patient with severe COVID-19.

The adipose tissue volumes of different fat components were assessed using a semi-automated segmentation method, which is based on SIMENS Healthineers and includes the parameters listed below. The typical segmentation results are illustrated in Figure 1.

- The volume of cardiac adipose tissue (CAT) in mm^3^ was determined through automated AI-based segmentation of the cardiac region, utilizing thresholding centered on the density range specific to adipose tissue values (−190 to −30 HU).
- For the quantification and normalization of abdominal visceral and subcutaneous fat, the first slice at the thoracoabdominal level, where the bases of the lungs were no longer visible, was selected; the last slice was the sixth slice following the first.
- In this stack of images, visceral adipose tissue (VAT) and subcutaneous adipose tissue (SAT) were performed using a semi-automated method. Automated segmentation of visceral and subcutaneous fat was performed centrally on the density range of adipose tissue values (−190 to −30 HU). Then, the external and internal contours of the abdominal wall were drawn to separate the VAT and SAT. The VAT and SAT volumes were measured automatically in mm^3^.

### 2.6 Statistical analysis

Data were analyzed using SPSS software for Windows (version 25.0, IBM Corp, Armonk, NY, United States). Numeric variables are presented as medians with interquartile ranges (IQR), while qualitative variables are shown as frequencies and percentages. Group differences in summary statistics were assessed using the Mann–Whitney U test, chi-square test, or Fisher’s exact test, as appropriate. Spearman or Pearson correlation coefficients were employed to evaluate the relationships between various adipose tissue biomarkers and between inflammatory factors, CT scores of pulmonary lesions, and PNI. For each group, all parameters that might affect in-hospital mortality were screened using univariate analysis, and multivariate logistic regression models were constructed according to the clinical relevance, univariate statistical associations, and the absence of collinearity. Receiver-operating characteristic (ROC) curves were constructed to assess the diagnostic value of these models.

## 3 Results

### 3.1 Study population

**Figure 2:**
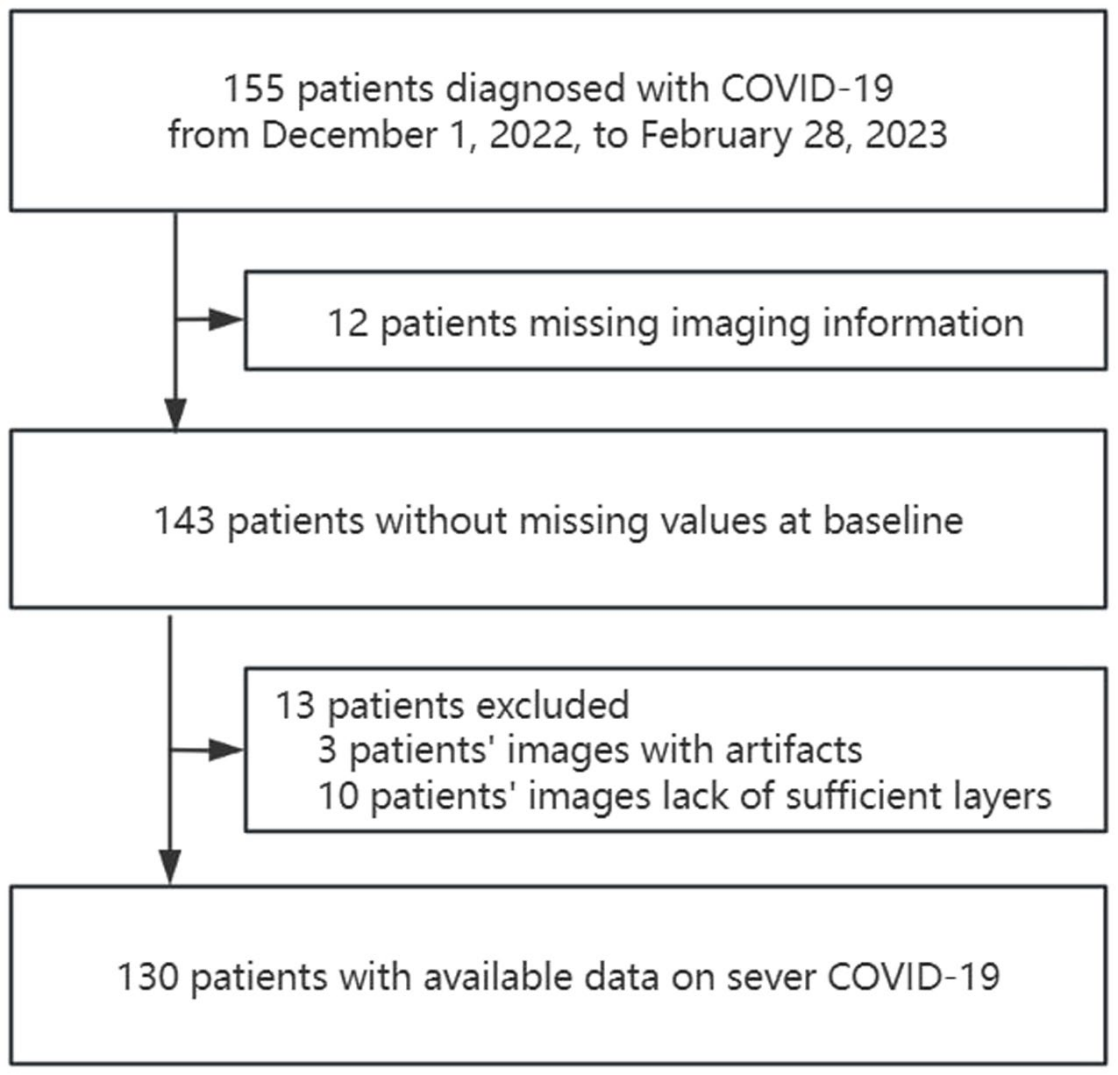
Flowchart describing patient selection.

The study identified 155 patients who were diagnosed with COVID-19 from December 1, 2022, to February 28, 2023. Of these, 130 patients met the inclusion criteria. (Figure 2).

The median age of the patients was 80.5 years old, and most were women (68%). Of the patients, 62 (48%) patients survived, while 68 (52%) patients died; the average length of stay from admission to death in the death group was 14.06 days, and 73 (56%) patients were treated with non-invasive ventilation. Most of the patients were with comorbidities, 80 (62%) patients had hypertension, 45 (35%) patients had diabetes, 35 (27%) patients had a stroke, 12 (9%) patients had a tumor, 33 (25%) patients had coronary heart disease, 25 (19%) patients had fibrillation, 26 (20%) patients had chronic lung disease and 35 (27%) patients had chronic nephropathy.

The baseline characteristics, as well as the imaging and biological data of the patients, are shown in Table 2. In the death group, more patients with fibrillation (24% vs. 15%, *p* = 0.002), nephropathy (47% vs. 5%, *p* < 0.001), and more severe pulmonary lesions based on CT score (20 vs. 15, *p* = 0.011) comparing with survival group, but the less diabetes patients were found in death group (25% vs. 45%, *p* = 0.016). In terms of laboratory tests, the patients of death group had higher LDH (389 vs. 268 U/L, *p* = 0.002), D-dimer (800.5 vs. 180.0 μmol/L, *p* < 0.001), total bilirubin (13.3 vs. 10.6 μmol/L, *p* = 0.026), creatinine (118.65 vs. 69.75 μmol/L, *p* < 0.001), WBC (10.3 vs. 6.9 10^9^/L, *p* = 0.002), neutrophil counts (9.205 vs. 5.915 10^9^/L, *p* < 0.001), BUN (14.955 vs. 7.095 mmol/L, *p* < 0.001), CRP (138.845 vs. 61.3 mg/dL, *p* < 0.001), IL-6 (88.2 vs. 19.78 pg./mL, *p* = 0.017); and had a lower lymphocyte counts (0.515 vs. 0.76 10^9^/L, *p* = 0.011), eGFR (47.47 vs. 83.48 mL/min, *p* < 0.001) and albumin (30.8 vs. 34.05 g/L, *p* < 0.001). As for adipose tissue imaging biomarkers, only SAT was significantly higher in the survival group (423.29 vs. 542.23 10^4^ mm^3^, *p* = 0.002) compared with the death group. The PNI was higher (33.425 vs. 37.8, *p* < 0.001) in the survival group.

**Table 2:** Baseline characteristics of the included population and comparison between deaths and survivals.

	Total (*n* = 130)	Death (*n* = 68)	Survivor (*n* = 62)	*p*-value
Characteristics				
Age, years	80.50(73–87.25)	80.5(70.25–88)	80.5(74.00–86.25)	0.978
Male, %	42(32)	17(25)	25(40)	0.062
CT score of pulmonary lesions	18(11–23)	20(14.25–24)	15(10–22)	0.011*
Hypertension, %	80(62)	39(57)	41(66)	0.304
Diabetes, %	45(35)	17(25)	28(45)	0.016*
Stroke %	35(27)	21(31)	14(23)	0.286
Tumour, %	12(9)	8(12)	4(6)	0.296
Coronary heart disease, %	33(25)	18(26)	15(24)	0.766
Fibrillation, %	25(19)	16(24)	9(15)	0.002*
Chronic lung disease, %	26(20)	11(16)	15(24)	0.254
Chronic nephropathy, %	35(27)	32(47)	3(5)	<0.001*
Non-invasive ventilation, %	73(56)	22(32)	51(82)	<0.001*
Laboratory findings				
ALT, U/L	26(14–38)	25(16–37)	26(14–38)	0.073
AST, U/L	37(27–60.25)	50(30.25–74.5)	31(24–47)	0.05
LDH, U/L	313(238–449.75)	389(272–575.25)	268(213.75–348.75)	0.002*
CK-MB, U/L	13(9–21.5)	17(12–30)	10(8.5–15.5)	0.059
D-dimer, μmol/L	336(143–1,076)	800.5(290.5–1898)	180(96.25–478.5)	<0.001*
Total bilirubin, μmol/L	11.20(7.50–15.05)	13.30(8.23–18.33)	10.60(6.95–13.10)	0.026*
Creatinine count, μmol/L	85.05(60.95–145.45)	118.65(78.56–236.75)	69.75(53.83–89.3)	<0.001*
Neutrophil count, 10^9^/L	7.33(4.06–11.26)	9.205(5.45–12.87)	5.915(2.82–9.26)	<0.001*
Lymphocyte count, 10^9^/L	0.605(0.4–0.88)	0.515(0.33–0.78)	0.76(0.49–0.97)	0.011*
Platelet, 10^9^/L	154(105.75–212.5)	143(95.5–203.75)	167(112.75–228)	0.493
BUN, mmol/L	9.705(6.53–16.17)	14.955(8.78–24.59)	7.095(5.28–9.84)	<0.001*
eGFR, ml/min	66.64(35.53–87.05)	47.47(18.09–76.84)	83.48(62.25–90.84)	<0.001*
Albumin, g/L	32.6(29.93–35.7)	30.8(28.33–34.75)	34.05(31.2–36.7)	<0.001*
Globulin, g/L	27.35(23.4–30.93)	28(23.83–31.6)	26.8(22.9–30.47)	0.178
WBC count, 10^9^/L	8.6(5–12.83)	10.3(6.75–13.8)	6.9(4.1–10.625)	0.002*
CRP, mg/dL	87.835(30.23–162.6)	138.845(50.4–189.53)	61.3(13.53–115.2)	<0.001*
IL-6, pg./mL	43.335(15.62–137.44)	88.2(27.12–209.67)	19.78(10.25–73.53)	0.017*
PNI	36.225(32.55–39.3)	33.425(30.41–36.99)	37.8(34.6–40.4)	<0.001*
Adipose tissue imaging biomarkers				
CAT, 10^4^mm^3^	156.51(104.11–224.24)	147.78(89.16–232.90)	162.42(110.12–223.54)	0.362
VAT, 10^4^mm^3^	538.21(332.25–795.74)	547.915(274.71–901.60)	535.885(404.36–737.14)	0.903
SAT, 10^4^mm^3^	466.58(286.16–660.23)	423.29(195.98–588.82)	542.23(385.69–776.99)	0.002*

Data presented as n (%) or median (IQR). ALT, Alanine aminotransferase; AST, Aspartate aminotransferase; LDH, lactate dehydrogenase; BUN, blood urea nitrogen; CK-MB, Creatine Kinase-MB; GFR, glomerular filtration rate; WBC, White blood cell; CRP, C-reactive protein; IL-6, Interleukin-6; CAT, cardiac adipose tissue; VAT, visceral adipose tissue; SAT, subcutaneous adipose tissue; PNI, prognostic nutritional index; *p*-values indicate differences between survivors and non-survivors. ^*^p < 0.05 was considered statistically significant.

### 3.2 Association between clinical, biological, and radiological data and patient outcomes

Univariate analysis of the main clinical, biological, and radiological findings are detailed in Table 3.

**Table 3:** Univariate analysis of variables for in-hospital death of patients with severe COVID-19.

	Univariate analysis		
	OR	95% CI	*p*-value
CT score of pulmonary lesions	1.49	1.075–2.067	0.017^*^
Chronic lung disease	1.641	0.356–7.565	0.525
Nephropathy	17.48	4.989–61.257	<0.001^*^
BUN, mmol/L	3.167	2.093–4.791	<0.001^*^
LDH, U/L	2.318	1.611–3.337	<0.001^*^
Creatinine count, μmol/L	2.638	1.825–3.944	<0.001^*^
Total bilirubin, μmol/L	1.607	1.160–2.226	0.004^*^
eGFR, ml/mini	0.964	0.951–0.979	<0.001^*^
WBC count, 10^9^/L	1.677	1.207–2.331	0.002^*^
Lymphocyte count, 10^9^/L	0.671	0.488–0.922	0.014^*^
Albumin, g/L	0.533	0.38–0.748	<0.001^*^
CRP, mg/dL	1.01	1.002–1.018	0.017^*^
IL-6, pg./mL	2.098	1.476–2.982	<0.001^*^
SAT, 10^4^mm^3^	0.64	0.463–0.885	0.007^*^
PNI	0.544	0.387–0.764	<0.001^*^

^*^p < 0.05 was considered statistically significant.

In the univariate analysis shown in Table 3, nephropathy, BUN, lactate dehydrogenase (LDH), creatinine level, total bilirubin, estimated glomerular filtration rate (eGFR), WBC count, lymphocyte count, albumin, CRP, interleukin-6 (IL-6), SAT, and PNI were associated with the death of critically ill patients with COVID-19. The CT score of pulmonary lesions was also associated with mortality of severe COVID-19.

### 3.3 Relationships between adipose tissue biomarkers, inflammatory factors, CT scores of pulmonary lesions, and PNIs

As shown in Table 4, only PNI showed a negative correlation with the CT score of pulmonary lesions. SAT was negatively correlated with CRP and positively correlated with PNI.

**Table 4:** Correlation analysis of adipose tissue biomarkers, inflammatory factors, CT scores of pulmonary lesions, and PNI.

	CRP	WBC	VAT	CAT	SAT	PNI	CT scores
CRP	1						
WBC	0.233^**^	1					
VAT	−0.017	0.016	1				
CAT	−0.137	0.049	0.731^**^	1			
SAT	−0.284^**^	−0.005	0.490^**^	0.549^**^	1		
PNI	−0.168	−0.101	0.082	0.190^*^	0.228^**^	1	
CT scores	0.129	0.142	−0.168	−0.081	−0.140	−0.214^*^	1

^*^ and ^**^ mean significant correlation at *p* < 0.05 and extremely significant correlation at *p* < 0.001.

### 3.4 Model development and validation

**Figure 3:**
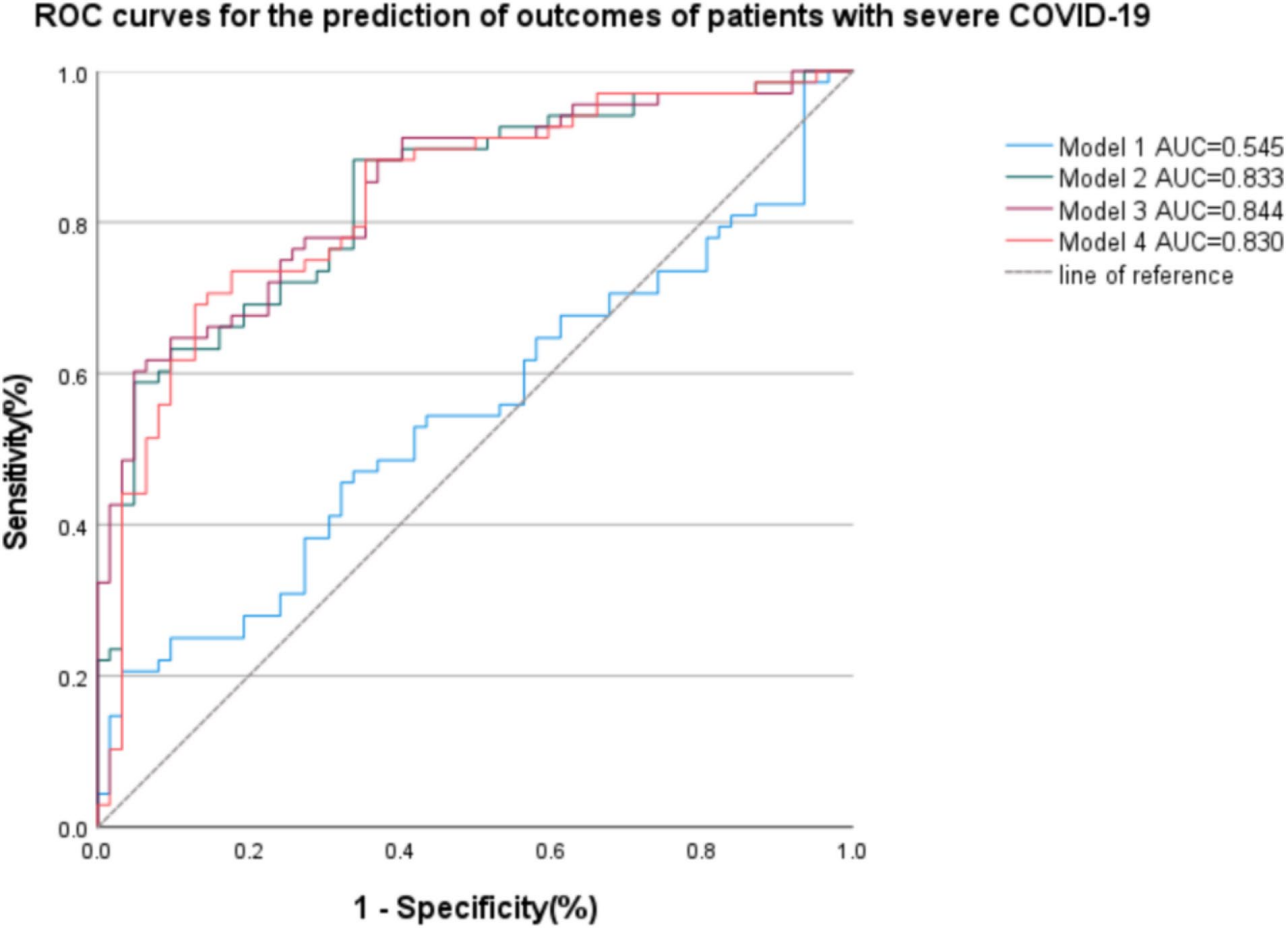
Receiver operating curves (ROC) of the four models are based on clinical, imaging, and biological data for the prediction of outcomes of patients with severe COVID-19. Model 1: age, Lymphocyte count, WBC, eGFR; AUC = 0.545. Model 2: age, Lymphocyte count, WBC, eGFR, PNI; AUC = 0.833. Model 3: age, Lymphocyte count, WBC, eGFR, SAT; AUC = 0.844. Model 4: age, Lymphocyte count, WBC, eGFR, SAT, PNI; AUC = 0.830.

**Figure 4:**
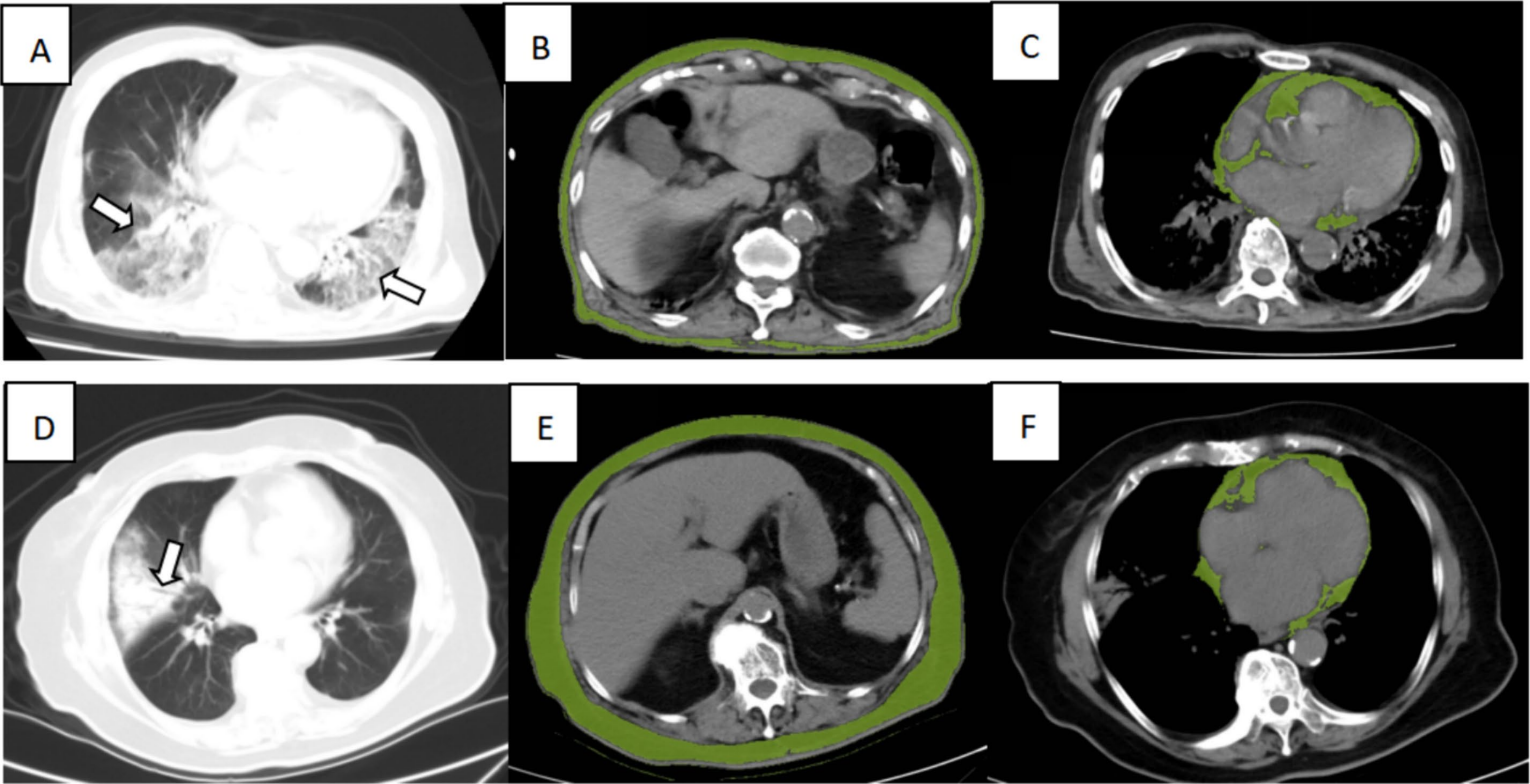
Illustration of CT imaging biomarkers assessed in two patients admitted for COVID-19 infection, including chest CT for lung severity and cardiac and subcutaneous abdominal adipose tissue. Top row: A 90-year-old man from the dead group presented extensive lung involvement with consolidations (**A**, arrows), with a low SAT (**B**, green overlay) and high VAT (**C**, green overlay). Bottom row: An 86-year-old woman from the surviving group presented consolidation of the right lung segment with air bronchogram (**D**, arrows), with a high SAT (**E**, green overlay), low CAT (**F**, green overlay).

After the determination of the relevant risk factors, four outcome prediction models were constructed, as shown in Table 5, with the ROC curves shown in Figure 3. Of these, the area under the curve (AUC) of Model 3, incorporating age, WBC count, eGFR, lymphocyte count, and SAT volume, was 0.844, which was higher than the AUCs of Model 1, which only included simple clinical and laboratory indicators and Model 2 in which the PNI values were included. A comparison of Model 3 with Model 4 indicated that the combination of PNI with SAT did not improve the accuracy of the prognostic model prediction. Patients with two different outcomes and their related imaging and test data are shown in Figure 4.

**Table 5:** Multivariate models for in-hospital death of patients with COVID-19.

	Multivariate models analysis		
	OR	95% CI	*p*-value
Model 1			
Age, years	1.023	0.987–1.060	0.212
Lymphocyte count, 10^9^/L	0.578	0.392–0.852	0.006*
WBC, 10^9^/L	1.655	1.108–2.471	0.014*
eGFR, ml/mini	0.400	0.262–0.611	<0.001*
Model 2			
Age, years	0.966	0.927–1.007	0.101
Lymphocyte count, 10^9^/L	0.689	0.452–1.049	0.083
WBC, 10^9^/L	1.562	1.026–2.377	0.037*
eGFR, ml/mini	0.381	0.242–0.600	<0.001*
PNI	0.594	0.423–0.835	0.003*
Model 3			
Age, years	0.973	0.936–1.011	0.164
Lymphocyte count, 10^9^/L	0.604	0.405–0.901	0.014*
WBC, 10^9^/L	1.621	1.076–2.441	0.021*
eGFR, ml/mini	0.412	0.267–0.634	<0.001*
SAT, 10^4^mm^3^	0.725	0.561–0.937	0.014*
Model 4			
Age, years	0.972	0.933–1.013	0.174
Lymphocyte count, 10^9^/L	0.700	0.456–1.075	0.103
WBC, 10^9^/L	1.540	1.005–2.359	0.047*
eGFR, ml/mini	0.395	0.250–0.624	<0.001*
SAT, 10^4^mm^3^	0.778	0.594–1.020	0.069
PNI	0.640	0.453–0.906	0.012*

^*^p < 0.05 was considered statistically significant.

## 4 Discussion

The purpose of this retrospective study was to explore the predictive effect of PNI combined with an evaluation of abdominal fat on the outcome of patients with severe COVID-19. The PNI was obtained from laboratory tests, including serum albumin level and total lymphocyte count. The fat compartments containing visceral, subcutaneous, and cardiac adipose tissue were obtained from the CT imaging of patients with COVID-19, which was for assessment of the severity of lung lesions. The study found that reduced SAT was an independent predictor of early mortality in patients with severe COVID-19. There was no significant relationship between VAT or CAT and mortality in patients with severe COVID-19.

Contrary to earlier studies (35–37) that explored the link between adipose tissue and mortality in COVID-19 patients, this study found no association between VAT and mortality among patients with severe COVID-19. This discrepancy may be due to the study’s sample size and the average age of participants. Previous studies (7, 23, 35–39) examined outcomes for patients with mild to moderate COVID-19, focusing on improvement, recovery, and progression to severe disease or death. In contrast, this study focused on outcomes among severely and critically ill patients, evaluating improvement, deterioration, or mortality. In previous studies (35–37), the median age of patients ranged from 60 to 70 years, whereas in this study, it was 80.5 years. But, in fact, the elderly patients are also a risk factor for severe COVID-19 (6). However, in this study, there was no significant difference between death and survival groups in terms of age. The possible reason is that the range of the total patients was only 14.25 years, but the amounts of younger patients with severe COVID-19 is too small, so it is hard to detect statistical differences.

Another factor could be the presence of comorbidities within the study population. A previous study (23) indicated that for patients with type 2 diabetes, CAT and VAT showed higher diagnostic efficacy compared to SAT, with no significant difference in mortality prediction for non-diabetic patients. Therefore, the relationship between CAT and the prognosis of severe COVID-19 patients may be more relevant to those with diabetes. Another study found that the increase of VAT and intermuscular adipose tissue (IMAT) is a kind of risk factor for death from COVID-19 (40). As far as we are concerned, it is very important that the patients of the study were mostly older patients with comorbidities or cachexia, but we cannot get the average BMI of this group. Indeed, total adipose tissue (TAT) and SAT are known risk factors with a U-shaped relationship, which means cachexia and obese patients are both at risk, whereas average obese patients are protected.

The study found that SAT was an independent predictor of death in patients with severe COVID-19. Although previous studies have explored the relationship between SAT and the outcomes of patients with COVID-19 (41), no models have been established using this index. To our knowledge, this is the first model to include the combination of SAT and clinical factors for the prediction of mortality in patients with severe COVID-19. The present study also established a separate clinical model for comparisons. Finally, the study concluded that the SAT + clinical factor model was more accurate in predicting prognosis than the model that only included clinical parameters. The PNI + SAT + clinical parameter model, however, did not show improved predictive accuracy. Linear regression analysis using PNI, SAT, CRP, and the CT score showed a positive correlation between PNI and SAT, which may be due to the interaction between the two, resulting in reduced diagnostic efficacy when the two values were combined. However, the predictive effectiveness showed slightly higher than PNI, though the PNI is still an important index to evaluate the nutritional status of patients, especially for the patients who are bedridden and unable to move, and which is more convenient to access.

An exploration of the relationship between SAT and inflammatory factors showed a significant negative correlation between SAT and CRP. A previous study (42) suggested that obesity-related chronic inflammation, along with dysfunctional mesenchymal stem cells and adipose-derived mesenchymal stem cells, could play key roles in exacerbating systemic inflammation. This, in turn, may contribute to cytokine storms and promote pulmonary fibrosis, leading to lung function failure, a hallmark of COVID-19. It is thus suggested that there may be a link between SAT and inflammation in patients with COVID-19, which warrants further exploration.

It has recently been found that the inflammatory response to SARS-CoV-2 infection may be linked with enforced immobility due to the illness, as well as reduced nutritional intake caused by taste disorders, olfactory loss, and frequent gastrointestinal involvement (43). In the present study, as shown in Table 3, SAT was found to be negatively correlated with CRP and positively correlated with PNI, suggesting that inflammation, subcutaneous adipose tissue, and malnutrition operate synergistically, increasing the likelihood of death in patients with severe COVID-19.

There are some limitations to this study. First, it was a single-central study, and multi-central studies with larger sample sizes are needed to verify the results. Because muti-central studies can collect data from diverse population groups, covering a wider range of geographic, social, economic, and cultural contexts, it helps to improve the generalizability of the findings. Selection bias due to specific settings, patient characteristics, or researcher preferences can be avoided. Second, all the CT images had a slice thickness of 5.0 mm, which could have reduced the precision of the volume measurements. A previous study has proposed that thinner slices can affect the measurement of the volume of VAT, but changes in SAT were not significant (44). In addition, because critically ill patients are mostly bedridden, it was difficult to assess the heights, weights, and body surface areas of these patients, preventing the standardization of these measurements. However, on the other hand, for critically ill patients with difficulty in collecting BMI, the measurement of body composition parameters such as fat or muscle using CT technology can indirectly reflect the patient’s weight (45). In this special case, imaging techniques and laboratory indicators can better predict his prognosis. The study originally planned to measure the volume of SAT and VAT between L1 and L2 vertebral bodies; however, the CT scan ranges of most patients are not sufficient to this standard, so the first image without lung bases was selected, and the following six slices were selected to limit the effect of slight differences from the depth of inspiration. Although all the patients underwent CT scans within 24 h of admission, it is very hard to confirm the exact time from disease occurrence to CT examination; the effect of its difference needs further study to confirm. The oxygen demand of patients with severe pneumonia changes dynamically throughout the process, so it is difficult to assess, which needs further research to verify it.

Many studies reported that patients after recovering from Long COVID syndrome, such as anxiety, depression, dizziness, chest pain, and so on (46–50). It could become the focus of further research, and we plan to follow up with patients to explore whether they have persistent long COVID symptoms such as survivors, disturbance, and sleep difficulty and to explore the relationship between the severity of the COVID-19 disease and the Long COVID syndrome. Although there is a study pointing out that the slicer thickness of CT imaging would not affect the volumetry of adipose tissue (43), we still recommend thinner slice CT or high-resolution CT scans for a more accurate measurement of fat volume. More clinical data, such as saturation, respiratory rate, blood pressure, etc., which are more relevant predictors of the outcome of patients, should be considered and recorded.

## 5 Conclusion

In summary, the findings showed that the prognosis of patients with severe COVID-19 was significantly correlated with inflammatory indicators such as WBC counts and CRP. Comorbidities, such as diabetes and nephropathy, increase the likelihood of death. Both the CT score of pulmonary lesions and SAT were found to be linked to mortality; however, no significant associations between CAT and VAT and mortality were observed. In the models of prognostic prediction, it was found that the AUC of Model 3 (incorporating age, lymphocyte count, WBC, eGFR, and SAT) was the highest, and the combination of PNI and SAT did not improve the accuracy of the model prediction.

## Data Availability

The original contributions presented in the study are included in the article/Supplementary material, further inquiries can be directed to the corresponding author.
